# Supplementary material for: Preserving privacy and video quality through remote physiological signal removal
Source: Commun Eng. 2025 Apr 7;4:66. doi: 10.1038/s44172-025-00363-z (PMC11977227; doi:10.1038/s44172-025-00363-z)
Supplement: Supplementary file 2 — Supplementary Information [file 44172_2025_363_MOESM2_ESM.pdf]

Supplementary Information

Preserving Privacy and Video Quality through Remote Physiological Signal Removal

Supplementary Note 1

Performance of three modification methods (Gaussian Blur, Median Blur and Time-Averaging sliding) with different hyper-parameters.

Supplementary Table 1 Performance of Median Blurring (MB) and Gaussian Blurring (GB) methods with different kernel sizes for one subject (n=1) for all the rPPG estimation techniques (n=5) and for all the activities (n=4) over the entire length of the videos.

Blurring Method	Kernel Size (k)	Δbpm						MSE	OS	fps
		CHROM	POS	LGI	GREEN	ICA	AVG			
Gaussian Blur (GB)	3	14.41	11.965	11.9675	19.9525	21.335	15.926	23.79563	0.5	15.16
	5	17.795	16.77	19.225	22.44	24.8175	20.2095	30.69447	0.849765	14.88
	7	17.4925	16.4925	19.1625	21.9925	22.955	19.619	40.18712	0.375635	14.75
	9	17.495	16.255	18.665	22.365	22.1675	19.3895	46.75571	0.119526	14.54
Median Blur (MB)	3	14.5325	11.59	12.0275	19.7825	19.865	15.5595	21.75281	0.5	15.07
	5	17.6525	17.0075	19.38	21.7425	22.4125	19.639	38.68903	0.664503	14.53
	7	17.8625	17.23	18.9225	20.385	22.6	19.4	57.27876	0.495841	5.98
	9	18.5025	16.7075	18.58	20.1225	23.6	19.5025	69.7232	0.5	5.71

Supplementary Table 2 Performance of the Time-Averaging (TA-S) modification method for one subject (n=1) for all the rPPG estimation techniques (n=5) and for all the activities (n=4) over the entire length of the videos with varying window sizes.

Window Size (# frames)	Δbpm						MSE	OS
	CHROM	POS	LGI	GREEN	ICA	AVG		
5	22.5375	21.535	22.3275	26.5625	25.495	23.6915	60.39928423	0.5
10	23.0975	23.33	23.4475	27.65	25.43	24.591	85.46584428	0.370717469
15	24.91	25.0875	25.0025	30.2075	28.245	26.6905	100.8691517	0.397361672
20	29.3775	32.295	30.355	30.9875	31.8925	30.9815	110.2759191	0.620000841
25	26.0675	22.7125	25.395	30.3775	29.355	26.7815	118.6201712	0.268361968
30	24.97	23.53	25.0375	30.085	27.3475	26.194	126.0265806	0.171639232

Supplementary Note 2

Source data for the graphs in the manuscript.

Supplementary Table 3 |Δbpm| for each frame modification method in each rPPG estimation technique. The results are estimated for each subject (n=6) and each the activity (n=4) in the LGI-PPGI dataset, averaged for all rPPG estimation techniques (n=5) and over the entire length of the videos. The facial region without the eyes and mouth was adopted as the ROI.

Subject	Activity	NE	MB	GB	BB	TA-C	TA-S	AGN	AGN-L	SPN	PoN	PeN	SN
alex	gym	29.55	28.764	26.94	28.324	41.996	43.254	32.292	34.79	37.274	30.386	35.642	26.566
	resting	3.238	3.708	4.35	3.442	25.11	17.022	5.418	6.912	4.892	3.848	10.168	2.712
	rotation	5.18	6.584	5.99	5.944	21.47	21.068	13.874	8.59	16.106	6.066	12.088	7.262
	talk	14.746	15.264	14.184	14.264	24.894	23.24	23.792	23.394	31.49	16.022	30.134	20.572
angelo	resting	3.184	4.054	4.42	2.71	16.668	6.894	3.322	2.212	6.95	3.184	1.262	3.668
	rotation	6.038	5.944	6.958	6.716	27.676	9.526	7.34	7.27	9.358	6.96	8.806	6.398
	talk	6.99	6.74	6.662	6.558	13.298	12.246	15.932	10.394	20.72	6.914	13.176	7.55
cpi	gym	7.608	8.208	7.864	8.342	52.236	37.15	6.978	7.116	7.848	8.126	7.56	7.858
	resting	1.298	2.102	1.664	2.324	14.424	5.308	3.424	3.638	4.634	1.922	3.734	2.108
	rotation	5.194	5.336	5.854	4.978	16.62	11.552	6.916	5.992	6.266	5.712	5.618	5.782
	talk	19.982	19.254	19.736	19.516	33.412	27.114	22.256	23.18	25.964	21.474	17.828	20.366
david	gym	21.916	20.864	21.128	20.976	45.01	50.058	19.81	20.322	21.218	20.806	20.57	20.456
	resting	5.404	3.534	6.194	5.35	23.498	17.796	5.468	5.014	8.858	4.776	5.438	6.268
	rotation	8.21	8.352	9.008	9.31	24.108	20.884	9.092	9.242	14.35	10.058	10.43	7.126
	talk	14.014	14.122	14.018	13.712	24.308	19.642	27.19	20.932	23.002	14.478	22.81	15.29
felix	resting	5.334	5.83	5.444	5.176	7.936	4.73	4.824	5.124	5.428	4.786	4.778	5.114
	rotation	9.42	7.308	8.18	7.978	12.074	7.896	9.732	9.898	8.622	7.994	13.24	8.5
	talk	15.784	16.508	16.826	16.286	22.556	28.086	23.032	23.774	23.028	16.662	23.678	16.706
harun	gym	32.372	32.868	32.364	32.922	49.69	47.77	34.4	34.83	39.83	32.844	36.836	33.512
	resting	3.346	3.45	3.734	2.994	33.154	22.272	10.644	7.242	10.494	7.156	10.858	6.764
	rotation	11.082	10.626	10.73	10.696	27.91	24.47	12.744	15.668	16.824	12.112	14.098	12.652
	talk	12.682	12.964	12.76	13.334	32.064	26.38	19.706	21.816	23.742	14.172	24.542	16.046

Supplementary Table 4 Average frames per second (fps) for each modification method estimated by running an analysis on a MacBook Pro with Intel i5 and was averaged over 1,000 frames. The facial region without the eyes and mouth was adopted as the ROI.

Method	MB	GB	BB	AGN	SPN	PoN	SN	AGN-L	PeN	TA-C	TA-S
fps	15.01	15.42	8.05	4.41	9.19	2.32	7.29	6.19	9.00	10.11	10.06

Supplementary Table 5 Evaluation of different ROIs in terms of the average |Δbpm|, average MSE, and OS. The results are for one subject (n=1) in the LGI-PPGI dataset, for all the rPPG estimation techniques (n=5) and for all the activities (n=4) over the entire length of the videos.

ROI	BPM	MSE	OS
Full Frame	14.69	576.3	0.49
Face - (Eyes + Mouth)	14.72	153.16	0.95
Forehead + Cheeks	12.64	112.4	0.5
