# Supplementary material for: Preserving privacy and video quality through remote physiological signal removal
Source: Commun Eng. 2025 Apr 7;4:66. doi: 10.1038/s44172-025-00363-z (PMC11977227; doi:10.1038/s44172-025-00363-z)
Supplement: Supplementary file 5 — Reporting Summary [file 44172_2025_363_MOESM5_ESM.pdf]

Corresponding author(s): *Double-blind peer review submissions: write DBPR and your manuscript number here instead of author names.*

Lasing Reporting Summary

Nature Research wishes to improve the reproducibility of the work that we publish. This form is intended for publication with all accepted papers reporting claims of lasing and provides structure for consistency and transparency in reporting. Some list items might not apply to an individual manuscript, but all fields must be completed for clarity.

For further information on Nature Research policies, including our data availability policy, see Authors & Referees.

Experimental design

Please check: are the following details reported in the manuscript?

1. Threshold

Plots of device output power versus pump power over a wide range of values indicating a clear threshold

☐ Yes ☒ No — Our work does not involve use of any lasers.

2. Linewidth narrowing

Plots of spectral power density for the emission at pump powers below, around, and above the lasing threshold, indicating a clear linewidth narrowing at threshold

☐ Yes ☒ No — Our work does not involve use of any lasers.

Resolution of the spectrometer used to make spectral measurements

☐ Yes ☒ No — Our work does not involve use of any lasers.

3. Coherent emission

Measurements of the coherence and/or polarization of the emission

☐ Yes ☒ No — Our work does not involve use of any lasers.

4. Beam spatial profile

Image and/or measurement of the spatial shape and profile of the emission, showing a well-defined beam above threshold

☐ Yes ☒ No — Our work does not involve use of any lasers.

5. Operating conditions

Description of the laser and pumping conditions
Continuous-wave, pulsed, temperature of operation

☐ Yes ☒ No — Our work does not involve use of any lasers.

Threshold values provided as density values (e.g. W cm^{-2} or J cm^{-2}) taking into account the area of the device

☐ Yes ☒ No — Our work does not involve use of any lasers.

6. Alternative explanations

Reasoning as to why alternative explanations have been ruled out as responsible for the emission characteristics
e.g. amplified spontaneous, directional scattering; modification of fluorescence spectrum by the cavity

☐ Yes ☒ No — Our work does not involve use of any lasers.

7. Theoretical analysis

Theoretical analysis that ensures that the experimental values measured are realistic and reasonable
e.g. laser threshold, linewidth, cavity gain-loss, efficiency

☐ Yes ☒ No — Our work does not involve use of any lasers.

8. Statistics

Number of devices fabricated and tested

☐ Yes ☒ No — Our work does not involve use of any lasers.

Statistical analysis of the device performance and lifetime (time to failure)

☐ Yes ☒ No — Our work does not involve use of any lasers.
